# Technical aspects of single-port thoracoscopic surgery for lobectomy

**DOI:** 10.1186/1749-8090-7-50

**Published:** 2012-06-06

**Authors:** Chih-Hao Chen, Shih-Yi Lee, Ho Chang, Hung-Chang Liu, Chao-Hung Chen, Wen-Chien Huang

**Affiliations:** 1 Graduate Institute of Mechanical and Electrical Engineering, National Taipei University of Technology, Taipei City, Taiwan; 2 Department of Thoracic Surgery, Mackay Memorial Hospital, No. 92, Section 2, Chung Shan North Road, Taipei City, Taiwan; 3 Division of Pulmonary and Critical Care Medicine, Mackay Memorial Hospital, Taipei City, Taiwan; 4 Mackay Medicine, Nursing and Management College, Taipei City, Taiwan

**Keywords:** Thoracoscopy/VATS, Surgery

## Abstract

Thoracoscopic Surgery is in common use in routine surgical practice. With the advancement of the various techniques and instruments required, mini wounds and fewer thoracoports become practical in recent years. Here, we report our experience of performing lobectomy with radical lymph node dissection in 3 patients using regular straight endoscopic instruments. We demonstrate the feasibility of such techniques and discuss the key points of effectively performing the procedures. Because of the favorable outcomes, we encourage such procedures to be widely applied in surgical operations of various types.

## **Background**

Video assisted thoracoscopic surgery (VATS) is indicated for any thoracic procedure that would be aided by the use of a thoracoscope. At present, there is no clear definition of how small an effective endoscopic procedure may be. In the past, thoracic procedures have usually been performed through multiple port wounds, especially in complicated cases of cancer resection. Single- incision thoracoscopic surgery is also known as single-port thoracoscopic surgery or uni-portal thoracoscopic surgery. Single- incision thoracoscopic surgery grown increasingly popular in recent years. A variety of general thoracic surgery procedures can be accomplished by a single port wound instead of the multiple port wounds used previously. Certain modified tools are very helpful in the execution of single-port endoscopic surgery, such as reticulating instruments [1,2]. However, such a procedure is infrequently required in cancer surgery [3]. In general, a port wound is typically 1.5 to 2.5 cm. In certain circumstances, up to 3.5 cm may be necessary to pull a large specimen out, such as in the case of one or two lobes of lung tissues. Here, we report 3 cases of lung cancer. The three patients were treated with a single-incision approach for lobectomy and radical lymph node dissection. The technical aspects are discussed.

## **Case Presentation**

### **Case 1**

**Figure 1:**
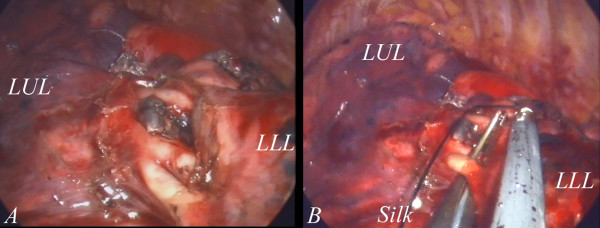
**The inter-lobar structure was exposed after adequate dissection.** The branches of the pulmonary arteries and inter-lobar lymph nodes are clearly seen.(**1A**) The vascular branch was looped with a right-angle clamp. A silk tie was placed for the lifting of the branch. (**1B**).

**Figure 2:**
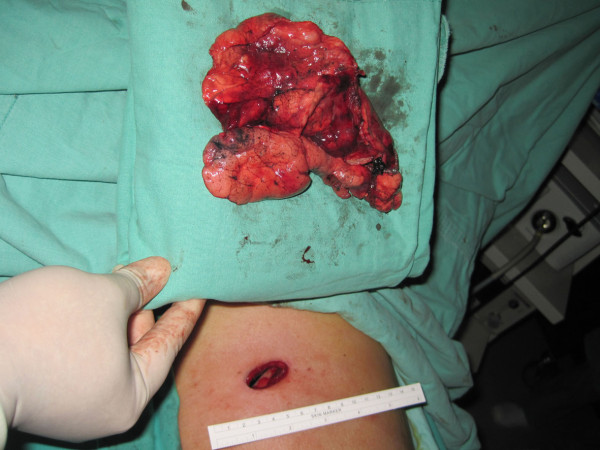
The enormous specimen of the left lower lobe together with the tumor can be adequately removed without destruction of the hard portion through a single port wound of 3.5 cm.

**Figure 3:**
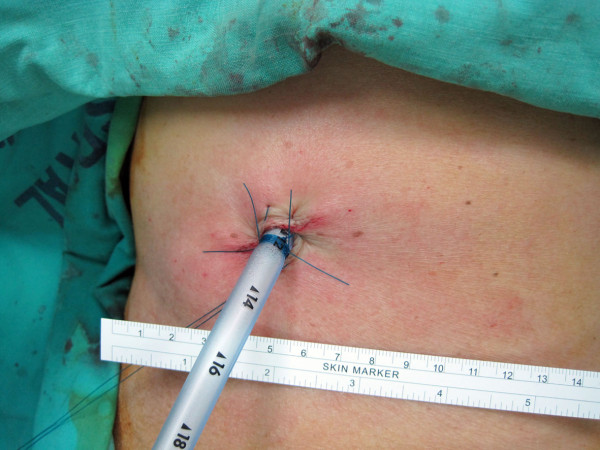
**The small wound is just small enough to place one chest tube.** There is no other wound.

A 67-year-old woman sought medical attention in our outpatient department and complained of mild chest discomfort. She reported that she had not had any systemic disease in the past and that she was a non-smoker. She also had not had any noticeable weight loss. Subsequent chest radiograph showed a faint opacity in left middle lung field. Therefore, she was admitted for further survey. Chest computed tomographic( CT) scan showed a lesion with features of air-bronchogram and a minimal solid component in the superior segment of the left lower lobe and a tiny nodule near the primary lesion. Preoperative survey, including sputum cytology, bronchoscopy and chest ultrasound, failed to yield a definitive diagnosis. We then proceed to a thoracoscopic procedure to obtain a definitive histologic diagnosis and decide the proper course of treatment. Initially**,** we used a single port of approximately 2.5 cm to perform a biopsy (wedge resection) of the lesion. During the procedure, a 5-mm rigid thoracoscope was used for viewing, along with a linear stapler and endoscopic grasping forceps for wedge resection. There was no reticulating instrument used in the procedure. The tumor was then removed with an endo-bag. After confirming the malignancy on frozen sectioning, we then proceeded to a radical resection of the lower lobe followed by radical lymph node dissection. The tools which were used during division of branches of the pulmonary artery and veins as well as the bronchus included a peanut sponge in the tip of a dissector, hook electrical cauterization, a long right-angle clamp, silk thread and staplers. The electrical cauterization hook was used to separate the fissure and then the peanut was used for blunt dissection to expose the inter-lobar structures. Once the vessels were exposed (Figure 1A), the long right-angle clamp was used to loop the vessels with silk.(Figure 1B) Then the silk tie was lifted to create a tunnel for the stapler to divide the branches of the artery and vein. The bronchus was divided as the last step. When the branches of the pulmonary artery, vein and the bronchus had been divided, the lower lobe was resected. Because the size of the lobe was too large to be removed from a 2.5-cm wound, the wound was slightly extended to 3.5 cm to allow the entire lobe to be pulled out into the endo-bag. (Figure 2 ) There were many small lymph nodes in St. 5, 7, 9 and 10. A harmonic scalpel and an endoscopic dissector used to dissect the lymph nodes. This whole procedure took approximately 2.5 hour to complete. At the end of the procedure, we placed one Fr.24 chest tube in the pleural cavity (Figure 3) and it was fixed in the single port wound. The estimated blood loss was approximately 50 ml, and forty-eight hours after the procedure, the chest tube was removed. Seventy-two hours after the procedure, the patient was discharged. Pathology showed two separate tumors, pT1aN0M0 and pT1bN0M0, which were both *in situ* adenocarcinomas (prior name : bronchioalveolar carcibnoma, BAC ) Lymph nodes were all negative for malignancy. Due to the multiple focal BACs, she is now on adjuvant chemotheraphy.

### **Case 2**

A 70-year-old woman complained of chest tightness. During her annual heath examination, a chest radiograph revealed an irregular opacity in the left lower lung field. She was then admitted for cancer screening. A CT scan uncovered a tumor in the superior segment of the left lower lobe of mediastinal lymphadenopathy, especially in the aortopulmonary window. Further investigation, including sputum cytology and percutaneous needle biopsy, failed to obtain a clear histology. She was then prepared for surgery in order to both obtain a diagnosis and identify the treatment course. During the operation, a 2.5-cm wound was initially made after the induction of general anesthesia. Using straight instruments to identify the tumor in the left lower lobe, a linear stapler was employed for wedge resection. The frozen section displayed adenocarcinoma. Because of the tumor’s malignant nature, we then proceeded to lobectomy and radical lymph node dissection. Before the procedure, we slightly extended the wound to 4 cm. The wound was protected with a plastic wound protector. The procedure took approximately 3 hours to complete. The estimated blood loss was 300 ml. After observation for 2 days, the chest tube was removed. Three days after the operation, the patient was discharged. The tumor was 2.5 cm in its greatest dimension. The station 5 (AP window) lymph nodes and hilar lymph nodes were positive for malignancy. She is currently on adjuvant chemo-radiation due to this pIIIA adenocarcinoma.

### **Case 3**

A 47-year-old man complained of productive cough for several months. He indicated that he had no past history of disease. He sought medical attention in the outpatient department. After a series of work-ups, he was found to have multiple small nodules in the bilateral lungs and mediastinal lymphadenopathy. Bronchoscopic findings were normal and washing cytology was also negative for malignancy. He was then treated surgically to confirm the diagnosis. Because the larger lesion was easily approached in the right middle lobe, we performed thoracoscopic surgery on the right side. Due to the more centralized location of the lesion, lobectomy of right middle lobe was performed. The final wound size was 3.5 cm in length. The estimated blood loss was less than 30 ml. The operative procedure elapsed time was 90 minutes. The endotracheal tube was removed immediately after the procedure. His chest tube was removed 2 days later and he was discharged one day after that. The final pathology report indicated adenocarcinoma *in situ*. Due to this *in situ* multiple focal adenocarcinoma, he is currently on chemotherapy.

### **Commentary**

Video-assisted thoracoscopic surgery through a single incision is a challenging technique for most thoracic surgeons. The transition from conventional thoracotomy to thoracoscopic surgery took a longer time than might have been expected. Currently, the transition from multi-portal thoracoscopic surgery to uni-portal thoracoscopic surgery is also taking a fair amount of time. It is reasonable that thoracic surgeons would want to know whether such a procedure is feasible, safe and has at least an equivalent oncological outcome as the previous procedures. This brief paper reports the feasibility and safety only. The long-term oncological outcome remains to be determined after more patients have undergone such procedures.

### **Conventional endoscopic multiport procedures**

**Figure 4:**
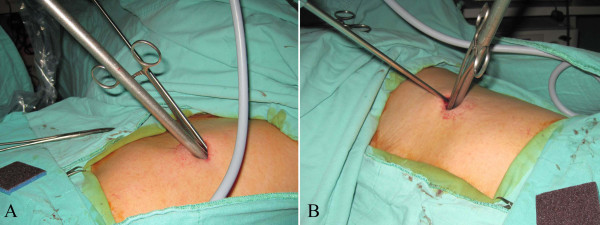
**A 2.5-cm wound for wedge resection of the lung is technically difficult using regular straight endoscopic instruments.** However, if an adequate port location is selected, such procedure is still safe and feasible (**4A** and **4B**).

Wedge resection of the suspicious lesion in the lung can be easily achieved using conventional methods, including multiple port wounds and multiple instruments. With the assistance of reticulating instruments, such a procedure may be made easier [1,2]. Employing instruments from different angles helps create the sense of a three-dimensional working environment. When necessary, more port wounds may be required for retraction of other tissues to assist the dissection. With multiple straight instruments through a single port wound, the procedure may be difficult but nevertheless, still feasible. (Figure 4A and 4B) A major difficulty is the very limited endoscopic field-of-view afforded the surgeon. In the procedure, we used a 5 mm endoscope with a 30-degree viewing angle. The field-of-view is still limited because the other instruments still occupy the viewing field, especially in the case of large tools such as stapler.

### **Localization of neoplasm**

A potential problem in the diagnostic procedure is the lack of tactile sensation. Especially in the setting of bronchoalveolar carcinoma with air-bronchogram, it may sometimes be difficult to definitely confirm the location of the neoplasm in the absence of pleural dimpling or if the solid component is tiny. A CT–guided needle localization by the radiologist may help in such an event. In our case, we did not perform needle localization because the neoplasm was identified. Delicate tactile exploration to find a tiny or soft neoplasm using a variety of instruments is sometimes possible for experienced surgeons. In fact, at times, the index finger is used to palpate the tumor through the small wound in order to avoid uncertainty. Whatever the techniques applied, it is crucial to confirm the tumor location prior to the procedure. Without an accurate preoperative localization, there will be uncertainty during the course of the endoscopic procedure.

### **Adequate position of the single port wound**

**Figure 5:**
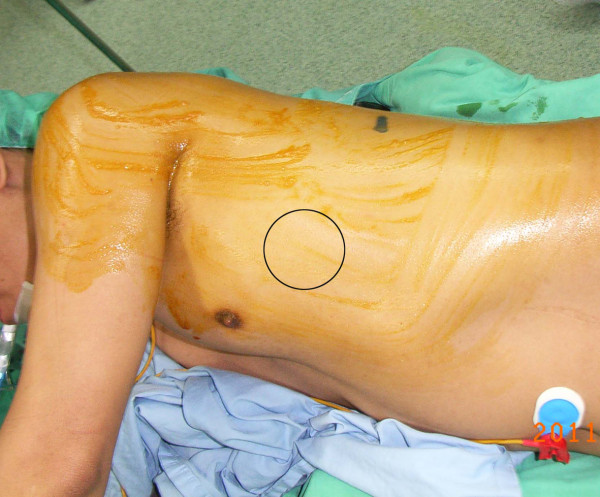
**When using single incision thoracoscopic surgery, an anterolateral port wound ( 4th to 6th intercostal space near the anetrior axillary line) is more appropriate in most circumstances.** The major reason is that there is a wider intercostal space than that in a posterolateral incision. With very limited intercostal space, a single incision procedure is very difficult.

Appropriate instruments and the position of the port wounds are also important. With regular straight (non-cross) instruments, the neoplasm cannot be resected if there is an inconvenient location of the port wound. Therefore, preoperative planning of the exact location of the single port wound to be created is essential. For lower lobe resection, the design of the single wound is in the 5th or 6th intercostal space between the anterior and mid-axillary lines. Dissection is generally easier when the wound is more anterior, because the intercostal space is wider, but lymph node dissection may be difficult, especially at station 7. (Figure 5) In the mid-axillary line, the separation of the inter-lobar structures is more difficult. Therefore, based on our experience, a wound in the 5th intercostal space near the anterior axillary line is better for both lower lobe resection and lymph node dissection.

### **The necessity of the trocar**

**Figure 6:**
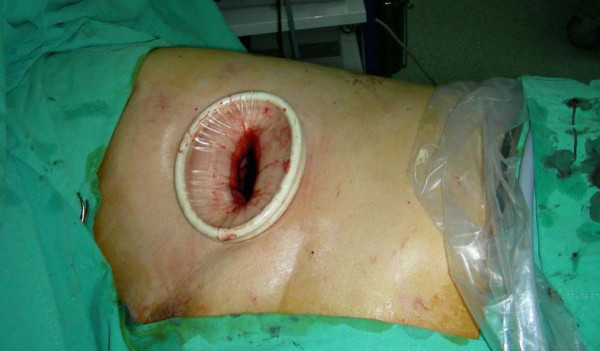
**A plastic wound protector is better than the use of conventional trocar.** The cylindrical lumen of a trocar limits the effectiveness of straight endoscopic instruments. With the wound protector, the manipulation of instruments is made easier because a cross-placement of the instruments becomes possible.

The need of using a trocar in such a procedure is controversial. When using a regular thoracoport, it is not possible to place multiple straight instruments within the trocar, which renders the procedure impossible. There are two solutions to this problem. One is to use a multi-access trocar, as previously described [4]. Another choice is to abandon the trocar. For example, we have used a plastic wound protector instead. (Figure 6) The plastic wound protector does not interfere with the cross placement of the instruments and affords maximal working space for both resection and identification of the neoplasm. Using multiple rigid trocar in a single wound seem to be unnecessary [2].

### **Division of fissures, branches of pulmonary vessels and bronchus**

**Figure 7:**
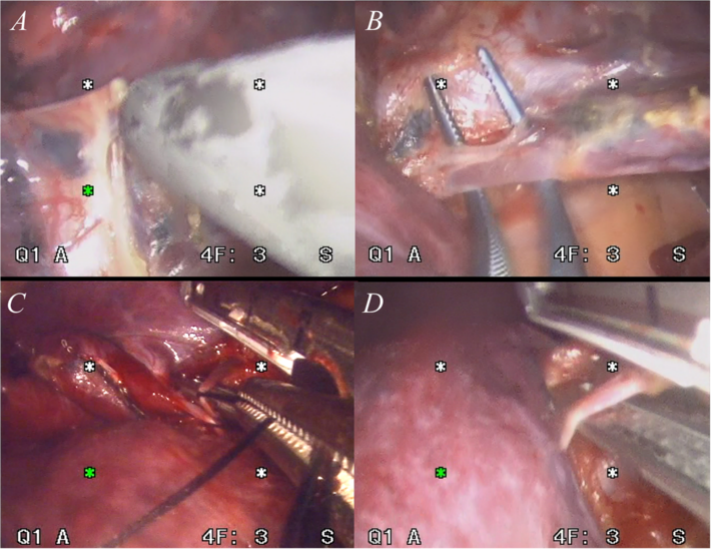
**Before starting to dissect the interlobar structure, the fissure must be separated.** In this case, we used a hook-shaped electrical cautery combined with blunt dissection with a peanut sponge to open the fissure(**7A**). After exposing the hilar vascular branches, a right angle clamp was used to loop the branch(**7B**) and then a silk tie was placed in order to exert traction the small branch(**7C**). With gentle traction of the branch, the stapler can be safely placed so as to separate the vessels. Surgeons have to make sure of the appropriateness of the relative position of the vessels and the stapler to allow a safe procedure(**7D**).

The steps to divide the inter-lobar fissure are similar to those in conventional endoscopic surgery. When an incomplete fissure makes an individual lobe difficult to separate, we usually try to approach the hilum first. If the vascular structures are safe, a curved clamp is used to create a tunnel above the inter-lobar vessels and bronchus that goes to the opposite side of the lung. Then a stapler is used to divide the individual lobe. When the fissure is obvious, we employ a hook electrical cautery to separate the fissure all the way to the inter-lobar structures. (Figure 7A) Then blunt dissection, such as with a peanut sponge, is used to identify the branches of the pulmonary vein and artery. When the branches of the vessels are visible, we use a long right-angle clamp to loop the vessels (Figure 7B), then the branches are looped with a silk tie. (Figure 7C) Minimal traction on the silk tie is sufficient to lift the vascular branch. Then a stapler may be safely placed across the vascular branch that is to be divided. (Figure 7D) Traction with a silk tie is very helpful in this step because placement of the stapler in the proper position without such counter-traction is very difficult. This was found to be the most time-consuming step. On occasion, many attempts were necessary due to an inadequate viewing field and/or wrong angle of approach. Once the view is very clear and the vascular branch is safe to divide, we proceed to fire the stapler. Making a mistake at this step results in excessive bleeding. All the branches were divided with similar techniques. The only difference was the angle of approach of each vessels. The bronchus was divided after all the vascular branches had been separated. The lymph nodes at each station were then checked. A harmonic scalpel was typically used to dissect the lymph nodes and the nodes were removed into a bag.

### **The minimum effective wound size**

**Figure 8:**
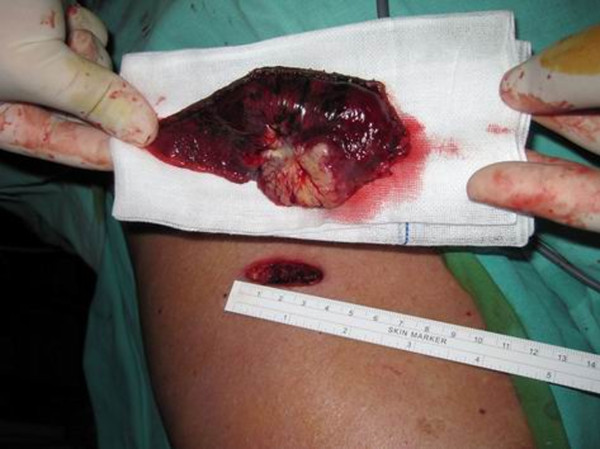
**The minimal requirement of a given wound size usually depends on the solid component of the tumor.** In order not to deform the tumor, the size of the wound is usually slightly larger than the solid portion. In this case, the shape is disk-like and approximately 3.5 cm. The wound is thus 3.7 cm and the tumor was removed in an endo-bag without any squeezing of the neoplasm.

A frequently asked question is the optimum final size of the wound. When a large specimen needs to be pulled out, is it possible to pull it out through a tiny port wound? According to our limited experience, the final size of the port wound in single-port VATS depends on the solid portion of the neoplasm (Figure 8) rather than the overall size. For large but very soft tissues, such as normal lung tissue or a specimen of primary spontaneous pneumothorax, they can be easily and gently retracted out into the endo-bag through a tiny wound [5]. The solid part, however, cannot be altered by pressure, as deformation may lead to confusion on the part of the pathologist upon microscopic examination. Therefore, in order not to rupture it, the wound may be made slightly larger than the size of the solid component. Sometimes lubrication may be needed between the bag, tumor and wound. In the cases we have reported, the solid portion is approximately 2.5 to 3 cm. A 3.5-cm wound allows the final extraction into an endo-bag.

The hospital stay was 3 days. The stay in the intensive care unit was 12 hours for 2 patients and the third patient was brought to the general ward. Postoperative pain was only minimal without the need of an epidural or intravenous patient-controlled anesthesia. The maximal visual analogue score for pain was 4, 4, and 5 for the three patients. Refraining from the use of a rib retractor during the operation is thought be an important means of decreasing the acute pain which can occur after thoracic surgery. The patients described here experienced only minimal intercostal neuralgia. In addition to not using a rib retractor, a smaller wound size also helps to alleviate acute wound pain. A shorter stay in both hospital and ICU is the expected outcome.

## **Conclusion**

Single-port thoracoscopic lobectomy with radical lymph node dissection is an alternative approach to conventional thoracoscopic lobectomy in lung cancer treatment. Although technically plausible and feasible, the issues of patient acceptability, the cosmetic and oncologic results, and ultimately cost-effectiveness, remain to be determined in the future using randomized-controlled trials and long-term follow-up.

## **Competing interests**

The authors declare that they have no competing interests.

## **Author's contributions**

CCH performed the operations and wrote the manuscript. LSY helped primary care of the patients and assist the data collection. HC, WCH and HCL helped revised the manuscript and provided technical support in the operation. CHC provided technical support. All authors read and approved the final manuscript.

## **Consent**

Written informed consent was obtained from the patient for publication of this report and any accompanying images.
